# Association between One-Hour Post-Load Plasma Glucose Levels and Vascular Stiffness in Essential Hypertension

**DOI:** 10.1371/journal.pone.0044470

**Published:** 2012-09-13

**Authors:** Angela Sciacqua, Raffaele Maio, Sofia Miceli, Alessandra Pascale, Giuseppe Carullo, Nadia Grillo, Franco Arturi, Giorgio Sesti, Francesco Perticone

**Affiliations:** Department of Medical and Surgical Sciences, University *Magna Græcia* of Catanzaro, Catanzaro, Italy; Scientific Directorate, Bambino Hospital, Italy

## Abstract

**Objectives:**

Pulse wave velocity (PWV) is a surrogate end-point for cardiovascular morbidity and mortality. A plasma glucose value ≥155 mg/dl for the 1-hour post-load plasma glucose during an oral glucose tolerance test (OGTT) is able to identify subjects with normal glucose tolerance (NGT) at high-risk for type-2 diabetes (T2D) and for subclinical organ damage. Thus, we addressed the question if 1-hour post-load plasma glucose levels, affects PWV and its central hemodynamic correlates, as augmentation pressure (AP) and augmentation index (AI).

**Methods:**

We enrolled 584 newly diagnosed hypertensives. All patients underwent OGTT and measurements of PWV, AP and AI. Insulin sensitivity was assessed by Matsuda-index.

**Results:**

Among participants, 424 were NGT and 160 had impaired glucose tolerance (IGT). Of 424 NGT, 278 had 1-h post-load plasma glucose <155 mg/dl (NGT<155) and 146 had 1-h post-load plasma glucose ≥155 mg/dl (NGT≥155). NGT≥155 had a worse insulin sensitivity and higher hs-CRP than NGT<155, similar to IGT subjects. In addition, NGT ≥155 in comparison with NGT<155 had higher central systolic blood pressure (134±12 vs 131±10 mmHg), as well as PWV (8.4±3.7 vs 6.7±1.7 m/s), AP (12.5±7.1 vs 9.8±5.7 mmHg) and AI (29.4±11.9 vs 25.1±12.4%), and similar to IGT. At multiple regression analysis, 1-h post-load plasma glucose resulted the major determinant of all indices of vascular stiffness.

**Conclusion:**

Hypertensive NGT≥155 subjects, compared with NGT<155, have higher PWV and its hemodynamic correlates that increase their cardiovascular risk profile.

## Introduction

Abnormal arterial stiffness, which usually develops with aging, is associated with increased risk for various adverse outcomes, including cardiovascular disease [1], [2], stroke [2], [3], and renal disease [4]. Arterial stiffness also increases in subjects with traditional cardiovascular risk factors suggesting, in addition to a background effect of aging per se, the effect of the exposure of the vessel wall to the cardiovascular risk factors, including hypertension, obesity, impaired glucose tolerance (IGT), and dyslipidemia [5]. In addition, there are some evidences demonstrating that arterial stiffness is associated with an important disparity between peripheral and aortic blood pressure (BP); particularly, several evidences suggest that aortic BP and its indices, such as augmentation pressure (AP) and augmentation index (AI), correlate more closely with intermediate markers of cardiovascular risk than brachial BP [6].

On the other hand, it’s known that type-2 diabetes mellitus (T2D) is an independent risk factor for heart failure even in absence of coronary artery disease or hypertension [7]. In addition, subjects with IGT and/or impaired fasting glucose (IFG) are characterized by an unfavorable cardiovascular risk profile [8].

Although normoglucose tolerant (NGT) subjects are considered at very low cardiovascular risk, recently a cutoff point of 155 mg/dl for the one-hour (1-h) post-load plasma glucose, during an oral glucose tolerance test (OGTT), was able to identify NGT subjects at high risk for T2D [9]. Moreover, 1-hour post-load plasma glucose value ≥155 mg/dl is strongly associated with different subclinical organ damages [10]–[13] that are independent predictors for cardiovascular events [7], [8], [14].

Taken together, we designed this study to address the question if glucose tolerance status, and in particular 1-h post-load plasma glucose levels, may affect arterial stiffness, evaluated by using applanation tonometry, in a group of never treated hypertensive Caucasian subjects.

## Methods

### Study Population

The study group consisted of 584 uncomplicated hypertensive outpatients, 322 men and 262 women aged 35–65 years (mean±SD = 46.5±9.8), participating to the CAtanzaro MEtabolic RIsk factors Study (CATAMERIS). All patients were Caucasian and underwent physical examination and review of their medical history. Causes of secondary hypertension were excluded by appropriate clinical and biochemical tests. Other exclusion criteria were history or clinical evidence of coronary and valvular heart disease, congestive heart failure, hyperlipidemia, peripheral vascular disease, chronic gastrointestinal diseases associated with malabsorption, chronic pancreatitis, history of any malignant disease, history of alcohol or drug abuse, liver or kidney failure and treatments able to modify glucose metabolism. No patient had ever been treated with antihypertensive drugs. All subjects underwent anthropometrical evaluation: weight, height, and body mass index (BMI).

After 12-h fasting, a 75 g OGTT was performed with 0, 30, 60, 90 and 120 minutes sampling for plasma glucose and insulin. Glucose tolerance status was defined on the basis of OGTT using the World Health Organization (WHO) criteria. Insulin sensitivity was evaluated using the Matsuda index [insulin sensitivity index (ISI)], calculated as follows: 10,000/square root of [fasting glucose (millimoles per liter)×fasting insulin (milliunits per liter)]*[mean glucose * mean insulin during OGTT]. The Matsuda index is strongly related to euglycemic hyperinsulinemic clamp that represents the gold standard test for measuring insulin sensitivity [15]. According to NCEP-ATPIII criteria, we also evaluated the presence/absence of metabolic syndrome (MS). The Ethical Committee approved the protocol and informed written consent was obtained from all participants. All the investigations were performed in accordance with the principles of the Declaration of Helsinki.

### Blood Pressure Measurements

Readings of clinic blood pressure (BP) were obtained in the left arm of the supine patients, after 5 min of quiet rest, with a mercury sphygmomanometer. Minimum three BP readings were taken on three separate occasions at least 2 weeks apart. Systolic and diastolic BP was recorded at the first appearance (phase I) and the disappearance (phase V) of Korotkoff sounds. Baseline BP values were the average of the last two of the three consecutive measurements obtained at intervals of 3 minutes. Patients with a clinic systolic BP (SBP) >140 mmHg and/or diastolic BP (DBP) >90 mmHg were defined as hypertensive.

### Laboratory Determinations

Plasma glucose was measured by the glucose oxidation method (Beckman Glucose Analyzer II; Beckman Instruments, Milan, Italy). Triglyceride, total, low- (LDL) and high-density lipoprotein (HDL) cholesterol concentrations were measured by enzymatic methods (Roche Diagnostics GmbH, Mannheim, Germany). Plasma insulin concentration was determined by a chemiluminescence-based assay (Roche Diagnostics).

### Arterial Wave Reflection and Central BP Measurements

These measurements were obtained by a validated system (Sphygmocor™; AtCor Medical, Sydney, Australia) that employs high-fidelity applanation tonometry (Millar) and appropriate computer software for the analysis of pressure wave (Sphygmocor™). Pressure calibration was obtained through automatically, non-invasively recorded supine brachial artery BP of the dominant arm after a 30-minute rest (Dinamap Compact T; Johnson & Johnson Medical Ltd, Newport, UK). BP was measured five times over 10 minutes and the mean of the last three measurements were taken for calibration. Pressure wave recording was performed at the radial artery of the dominant arm with the wrist softly hyperextended, and it is the average of single pressure waves recorded consecutively for eight seconds. Pressure wave recordings were accepted only if variation of peak and bottom pressures of single pressure waves was <5%. The central pressure wave was automatically derived from the radial pressures by a built-in generalized transfer function. In addition, was also obtained pressure wave measurement at the right carotid artery, as it is well known that central AI may be more accurately derived from this vascular site [16]. Central waveforms were further analysed to identify the time to peak/shoulder of the first (T1) and second (T2) pressure wave components during systole. The pressure at the peak/shoulder of T1 was identified as outgoing pressure wave height (P1), the pressure at the peak/shoulder of T2 was identified as the reflected pressure wave height (P2), either absolutely or as percent of ejection duration. AP was defined as difference between P2–P1, and AI as [AP/pulse pressure (PP)] * 100. Aortic pulse wave velocity (PWV) was determined from carotid and femoral pressure waveforms. Carotid to femoral transit time (ΔT) was computed from the foot-to-foot time difference between carotid and femoral waveforms. The distance between the surface markings of the sternal notch and femoral artery was used to estimate the path length between the carotid and femoral arteries (L), and PWV computed as L/ΔT.

### Statistical Analysis

Analysis of variance (ANOVA) for clinical and biological data was performed to test the differences among groups, and the Bonferroni post hoc test for multiple comparisons was further performed. Chi-squared test was utilized for categorical variables. Correlational coefficients were calculated according to Pearson’s method. Linear regression analysis was performed to correlate PWV, AP and AI with the following covariates: age, BMI, SBP, DBP, cholesterol values, fasting and 1-h and 2-h post-load plasma glucose levels, fasting and 1-h and 2-h post-load insulin, Matsuda index and high sensitivity C reactive protein (hs-CRP). Subsequently, variables reaching statistical significance and gender, MS and smoking status, as dichotomic value, were inserted in a stepwise multivariate linear regression model to determine the independent predictors of PWV, AP and AI. Correlational analysis was performed for whole study population and according to the groups of glucose tolerance. Data are reported as mean±SD. Differences were assumed to be significant at P<0.05. All comparisons were performed using the statistical package SPSS 16.0 for Windows (SPSS Inc., Chicago, Illinois, USA).

## Results

### Study Population

Of 640 subjects examined by OGTT, 56 were diabetic and were not included in the present analysis. Thus, of 584 remaining subjects, 278 had 1-h post-load plasma glucose <155 mg/dl (NGT<155), 146 individuals had 1-h post-load plasma glucose ≥155 mg/dl (NGT≥155), and 160 were IGT. Table 1 shows the demographic, clinical and biochemical characteristics of the study groups.

**Table 1:** Anthropometric and biochemical characteristics of the study population according to glucose tolerance status.

	All	NGT<155	NGT≥155	IGT	*P*
Variables	(n = 584)	(n = 278)	(n = 146)	(n = 160)	
**Gender,** *male/female*	322/262	147/131	88/58	87/73	0.338*
**Age,** *years*	46.5±9.8	46.3±11.1	46.4±10.1	47.1±6.5	0.698
**BMI,** *Kg/m^2^*	28.5±3.9	28.3±4.2	28.6±3.8	29.1±3.5	0.122
**Fasting glucose,** *mg/dl*	93.8±10.9	90.5±9.2	95.2±9.1	98.3±13.1	<0.0001
**1-h glucose,** *mg/dl*	153.3±41.2	120.8±22.3	182.2±25.5	183.4±35.4	<0.0001
**2-h glucose,** *mg/dl*	122.7±29.2	103.9±19.1	117.5±15.3	160.1±15.7	<0.0001
**Fasting insulin,** *µU/ml*	12.2±5.9	10.4±4.1	13.3±5.8	14.5±7.4	<0.0001
**1-h insulin,** *µU/ml*	111.2±72.1	88.9±55.7	156.1±95.1	109.9±53.5	<0.0001
**2-h insulin,** *µU/ml*	94.4±65.9	60.5±35.5	108.3±61.9	138.5±77.1	<0.0001
**MATSUDA index/ISI**	68.6±40.7	86.5±42.7	54.8±31.2	50.9±31.6	<0.0001
**Total cholesterol,** *mg/dl*	206.1±37.1	205.5±36.7	202.2±34.1	210.5±39.7	0.138
**HDL-cholesterol,** *mg/dl*	49.3±11.7	51.1±12.1	47.4±10.2	48.0±11.8	0.002
**LDL-cholesterol,** *mg/dl*	130.3±33.3	129.4±32.4	128.2±33.2	133.7±34.9	0.294
**Triglyceride,** *mg/dl*	133.1±71.3	126.4±72.4	133.7±68.3	144.2±70.9	0.041
**MS,** *n (%)*	271(46.4)	99(35.6)	72(49.4)	100(62.5)	<0.0001*
**hs-CRP,** *mg/L*	2.9±1.8	2.2±1.4	3.4±1.8	3.6±1.9	<0.0001
**Current smokers,** *n(%)*	170(29.1)	84(30.2)	28(19.2)	48(30.0)	0.037*

There were no significant differences among groups for gender distribution (P = 0.338), age (P = 0.698), BMI (P = 0.122), total cholesterol (P = 0.138), and LDL-cholesterol (P = 0.294). On the contrary, from the first to the third group of glucose tolerance, HDL-cholesterol significantly decreased (P = 0.002), while triglyceride (P = 0.041). hs-CRP (P<0.0001), prevalence of MS (P<0.0001) significantly increased. The prevalence of smokers was significantly different among groups (P = 0.037). Obviously, a progressive increase of fasting, 1-h and 2-h post-load glucose, as well as fasting insulin, parallel to the worsening of glucose tolerance (P<0.0001), as confirmed by MATSUDA index/ISI. Moreover, NGT ≥155 had a significant reduced insulin-sensitivity (P<0.0001) and an increased hs-CRP values (P<0.0001), when compared with NGT<155, and a metabolic and inflammatory profile similar to IGT individuals.

### Hemodynamic Parameters

Peripheral and aortic hemodynamic parameters for the study population, according to glucose tolerance groups, are reported in table 2. There were no significant differences among groups for peripheral BP values, central diastolic BP and heart rate (P = 0.067). On the contrary, from the first to the third group of glucose tolerance, we observed a significant increase of aortic systolic BP (P = 0.003) and PP (P<0.0001). Similarly, all indices of vascular stiffness progressively increased from the first to third group (P<0.0001). Notably, NGT≥155 subjects showed AP, AI and PWV not significantly different from IGT.

### Correlational Analysis

A linear regression analysis was performed to test the correlation between AP, AI and PWV and different covariates in the whole study population and in the groups according to glucose tolerance status.

AP, in the whole population, was significantly correlated with 1-h post-load glucose (r = 0.411, P<0.0001), 2-h post-load glucose (r = 0.219, P<0.0001), age (r = 0.173, P<0.0001), hs-CRP (r = 0.162, P<0.0001), LDL-cholesterol (r = 0.085, P = 0.019), fasting glucose (r = 0.121, P = 0.002), systolic BP (r = 0.104, P = 0.006), and total cholesterol (r = 0.096, P = 0.010). In NGT<155 subjects, AP was significantly correlated with 1-h post-load glucose (r = 0.319, P<0.0001), age (r = 0.260, P<0.0001), 2-h post-load glucose (r = 0.137, P = 0.011), total cholesterol (r = 0.122, P = 0.021) and BMI (r = 0.118, P = 0.024). In NGT≥155 subjects, AP correlated only with 1-h post-load glucose (r = 0.433, P<0.0001). In IGT patients, AP was correlated with 1-h post-load glucose (r = 0.370, P<0.0001), systolic BP (r = 0.256, P = 0.001), age (r = 0.218, P = 0.003) hs-CRP (r = 0.137, P = 0.042), and 2-h post-load glucose (r = 0.132, P = 0.047).

AI, in the whole population, was significantly correlated with 1-h post-load glucose (r = 0.401, P<0.0001), age (r = 0.216, P<0.0001), 2-h post load glucose (r = 0.198, P<0.0001), BMI (r = 0.090, P = 0.015), fasting plasma glucose (r = 0.133, P = 0,001), LDL-cholesterol (r = 0.094, P = 0.011), hs-CRP (r = 0.121, P = 0.002), total cholesterol (r = 0.106, P = 0.005) and triglyceride (r = 0.075, P = 0.035), and 2-h post-load insulin (r = 0.099, P = 0.008). In NGT<155 subjects, AI was significantly correlated with 1-h post-load glucose (r = 0.288, P<0.0001), age (r = 0.266, P<0.0001), 2-h post-load glucose (r = 0.128, P = 0.016), BMI (r = 0.124, P = 0.018), total cholesterol (r = 0.116, P = 0.026) and fasting glucose (r = 0.105, P = 0.040). In NGT≥155 subjects, AI correlated with 1-h post-load glucose (r = 0.493, P<0.0001), SBP (r = 0.150, P = 0.035) and LDL-cholesterol (r = 0.143, P = 0.043). In IGT patients, AI was correlated with 1-h post-load glucose (r = 0.402, P<0.0001), age (r = 0.237, P = 0.001), BMI (r = 0.139, P = 0.040), fasting insulin (r = 0.196, P = 0.007, and 1-h post-load insulin insulin (r = 0.162, P = 0.020).

PWV, in the whole population, was significantly correlated with 1-h post-load glucose (r = 0.503, P<0.0001), hs-CRP (r = 0.294, P<0.0001), 2-h post load plasma insulin (r = 0.243, P<0.0001), 1-h post-load plasma insulin (r = 0.200, P<0.0001), 2-h post-load glucose (r = 0.212, P<0.0001), age (r = 0.171, P<0.0001), fasting insulin (r = 0.156, P<0.0001), fasting glucose (r = 0.215, P<0.0001), systolic BP (r = 0.101, P = 0.007), LDL-cholesterol (r = 0.097, P = 0.010), total cholesterol (r = 0.111, P = 0.004). In NGT<155 subjects, PWV was significantly correlated with 1-h post-load glucose (r = 0.328, P<0.0001), age (r = 0.229, P<0.0001), 2-h post-load glucose (r = 0.194, P = 0.001), hs-CRP (r = 0.177, P = 0.002), 1-h post-load insulin (r = 0.147, P = 0.007) and total cholesterol (r = 0.140, P = 0.010). In NGT≥155 subjects, PWV was significantly correlated with 1-h post-load glucose (r = 0.570, P<0.0001), hs-CRP (r = 0.282, P<0.0001), 2-h post-load insulin (r = 0.219, P = 0.004), age (r = 0.176, P = 0.017), SBP (r = 0.176, P = 0.017), fasting insulin (r = 0.160, P = 0.027), fasting plasma glucose (r = 0.160, P = 0.026), 1-h post-load insulin (r = 0.148, P = 0.037), and Matsuda (r = −0.191, P = 0.010). <0.0001), 2-h post-load insulin (r = 0.234, P = 0.022) and LDL-cholesterol (r = 0.221, P = 0.029). In IGT patients, PWV was correlated with 1-h post-load glucose (r = 0.480, P<0.0001), triglyceride (r = 0.249, P = 0.001) hs-CRP (r = 0.179, P = 0.012), 2-h post-load glucose (r = 0.138, P = 0.041), fasting glucose (r = 0.214, P = 0.003), total cholesterol (r = 0.173, P = 0.014) and age (r = 0.154, P = 0.026).

**Table 2:** Peripheral and aortic hemodynamic parameters of the study population according to glucose tolerance status.

	All	NGT<155	NGT≥155	IGT	*P*
Variables	(n = 584)	(n = 278)	(n = 146)	(n = 160)	
**Heart rate,** *bts/min*	69±9	70±9	69±9	68±8	0.067
**systolic** **BP,** *mmHg*	145±13	145±11	144±14	145±14	0.711
**diastolic** **BP,** *mmHg*	91±7	91±7	92±7	91±8	0.357
**mean** **BP,** *mmHg*	109±7	109±6	109±7	109±8	0.999
**PP,** *mmHg*	54±14	54±13	53±14	54±15	0.751
**c-systolicBP,** *mmHg*	133±11	131±10	134±12	134±10	0.003
**c-diastolic** **BP,** *mmHg*	92±6	92±5	93±5	92±6	0.143
**c-PP,** *mmHg*	40±11	38±10	42±13	42±10	<0.0001
**AP,** *mmHg*	11.4±6.5	9.8±5.7	12.5±7.1	13.1±6.5	<0.0001
**AI,** *%*	27.7±12.5	25.1±12.4	29.4±11.9	30.7±12.5	<0.0001
**PWV,** *m/s*	7.5±2.7	6.7±1.7	8.4±3.7	8.2±2.9	<0.0001

* χ^2^ test; NGT  =  normal glucose tolerance; IGT  =  impaired glucose tolerance; DM  =  diabetes mellitus; BMI  =  body mass index; ISI  =  insulin sensitivity index; MS  =  metabolic syndrome; hs-CRP  =  high sensitivity C reactive protein; BP  =  blood pressure; PP  =  pulse pressure; c = central; PWV  =  pulse wave velocity; AP  =  augmentation pressure; AI  =  augmentation index.

Thus, variables reaching statistical significance, and gender, MS and smoking status, as dichotomic values, were inserted in a stepwise multivariate linear regression model to determine the independent predictors of AP, AI and PWV (Tables 3, 4, and 5), respectively. This analysis was performed in the total population and according to glucose tolerance status. In whole population, 1-h post-load glucose and age were the independent predictors of AP, explaining a 16.9% (P<0.0001) and 1.0% (P = 0.008) of its variation, respectively. In NGT<155 subjects, 1-h post-load glucose was the main predictor of AP, accounting for a 10.2% of its variation (P<0.0001). Other independent predictors were age and BMI explaining another 2.9% (P = 0.002) and 1.3% (P = 0.036) of AP variation. In NGT≥155 subjects and IGT patients, 1-h post-load glucose was the independent predictor of AP, accounting for the 18.8% (P<0.0001) and 13.7% (P<0.0001) of its variation in the respective models (Table 3).

**Table 3:** Stepwise multiple regression analysis on augmentation pressure as dependent variable in whole study population and in NGT subjects with different glucose tolerance status.

	All		NGT<155		NGT≥155		IGT		
	*Partial R^2^ (%)*	*P*	*Partial R^2^ (%)*	*P*	*Partial R^2^ (%)*	*P*	*Partial R^2^ (%)*	*P*	
**1-h glucose,** *mg/dl*	16.9	<0.0001	10.2	<0.0001	18.8	<0.0001	13.7	<0.0001	
**Age,** *yrs*	1.0	0.008	2.9	0.002	–	–	–	–	
**BMI,** *Kg/m^2^*	–	–	1.3	0.036	–	–	–	–	
**systolic BP,** *mmHg*	–	–	–	–	–	–	4.5	0.004	
***Total R^2^ (%)***	17.9		14.4		18.8		18.2		

BMI  =  body mass index; BP  =  blood pressure.

**Table 4:** Stepwise multiple regression analysis on augmentation index as dependent variable in whole study population and in NGT subjects with different glucose tolerance status.

	All			NGT<155			NGT≥155			IGT		
	*Partial R^2^ (%)*	*P*	*Partial R^2^ (%)*		*P*	*Partial R^2^ (%)*		*P*	*Partial R^2^ (%)*		*P*	
**1-h glucose,** *mg/dl*	16.0	<0.0001	8.3		<0.0001	24.3		<0.0001	16.1		<0.0001	
**Age,** *yrs*	2.2	<0.0001	4.2		<0.0001	–		–	–		–	
**Fasting insulin,** *µU/ml*	1.1	0.004	–		–	–		–	4.5		0.003	
**BMI,** *Kg/m^2^*	0.7	0.023	1.3		0.035				–		–	
**Gender**	0.6	0.045	1.8		0.027	–		–	–		–	
***Total R^2^ (%)***	20.6		15.6			24.3			20.6			

BMI  =  body mass index.

**Table 5:** Stepwise multiple regression analysis on pulse wave velocity as dependent variable in whole study population and in NGT subjects with different glucose tolerance status.

	All			NGT<155			NGT≥155			IGT	
	*Partial R^2^ (%)*	*P*	*Partial R^2^ (%)*		*P*	*Partial R^2^ (%)*		*P*	*Partial R^2^ (%)*		*P*
**1-h glucose,** *mg/dl*	25.3	<0.0001	10.8		<0.0001	32.6		<0.0001	23.0		<0.0001
**hs-CRP,** *mg/l*	1.6	<0.0001	2.9		0.004	3.2		0.007	–		–
**2-h insulin,** *µU/ml*	0.9	0.006	–		–	4.6		0.002	–		–
**Age,** *yrs*	0.8	0.011	2.1		0.014	–		–	–		–
**2-h glucose,** *mg/dl*	0.8	0.014	–		–	–		–			
**Smoking**	–	–	–		–	–		–	6.3		<0.0001
**Total cholesterol,** *mg/dl*	–	–	–		–	–		–	2.0		0.036
***Total R^2^ (%)***	29.4		15.8			40.4			31.3		

hs-CRP  =  high sensitivity C-reactive protein.

1-h post-load glucose was retained as the first independent predictor of AI in the whole study population and in the groups of glucose tolerance, explaining a 16.0% (P<0.0001) in the whole population, 8.3% (P<0.0001) in NGT<155, 24.3% (P<0.0001) in NGT≥155, and 16.1% (P<0.0001) in IGT patients, respectively. In the whole population, also age, fasting insulin, BMI and gender entered in the final model that explain the 20.6% of its variation. In NGT<155 subjects, age, BMI and gender contribute to explain another 7.3% of AI variation. In IGT group, also fasting insulin entered in the final model explaining another 4.5% of its variation (Table 4).

Similarly, 1-h post-load glucose resulted the first independent predictor of PWV in the whole study population and in the groups of glucose tolerance, explaining the 25.3% (P<0.0001) in the whole population, 10.8% (P<0.0001) in NGT<155, 32.6% in NGT≥155 (P<0.0001), and 23% in IGT of its variation, respectively. In the whole population, hs-CRP, 2-h insulin, age and 2-h post-load glucose explain another 4.1% of PWV variation. In NGT<155 subjects, hs-CRP and age explain another 5.0% of its variation. In NGT≥155 subjects, hs-CRP and 2-h insulin were retained as independent predictors of PWV, explaining another 7.8% of its variation. In IGT group, smoking and total cholesterol explain another 8.3% of PWV variation (Table 5).

## Discussion

Results of this study, conducted in a cohort of never treated hypertensive and NGT patients, showed that the worsening of glucose tolerance was associated with an increase of aortic stiffness and its hemodynamic correlates, such as AP and AI. The clinical relevance of these data consists in the fact that all these parameters correlate more closely with intermediate markers of risk for subsequent cardiovascular events [6]. Similarly, it is well established that there is a continuous linear relationship between fasting and post-load plasma glucose concentrations and incident cardiovascular events, even if 2-hour post-load glycemia has been identified as the most predictive index of mortality [17]. These findings are supported by the fact that worsening of glucose tolerance contributes to the pathogenesis of atherosclerotic vascular disease more than fasting plasma glucose. In keeping with this, NGT are considered subjects at lower cardiovascular risk. However, results of present study clearly demonstrate that in NGT ≥155 subjects arterial stiffness, a surrogate end-point for cardiovascular disease, and aortic hemodynamic parameters are significantly higher when compared to NGT<155 subjects. In addition, stepwise multiple regression analysis retained 1-h post-load plasma glucose as the first independent predictor of PWV in all groups (Table 4). In particular, it explains the 25.5% in the whole population, 10.8% of PWV variation in NGT<155 and 32.6% in NGT≥155 subjects, respectively.

Previous published results demonstrated an association between glucose tolerance worsening and PWV [18], [19], but at this moment there are no data demonstrating this relationship between vascular stiffness and 1-h post-load plasma glucose in NGT subjects. Thus, our data consent to extend previous knowledge about the association between glucose tolerance and arterial stiffness, demonstrating that NGT≥155 subjects in comparison with NGT<155 have a worse metabolic and hemodynamic profile that contributes to increase their global cardiovascular risk. Another important finding obtained in this study is that NGT≥155 subjects have both hs-CRP and reduced insulin sensitivity levels similar to individuals with IGT that are considered at high risk for both T2D and CV disease [20]. The coexistence of an increased PWV and central hemodynamic parameters in these subjects contributes to further amplify their cardiovascular risk profile. In addition, our data consent to reconsider the concept that NGT subjects are a homogeneous group with a low cardiovascular risk. Nevertheless, the evidence that NGT≥155 have vascular and metabolic modifications confirms that the major pathogenetic mechanism promoting organ damage may be considered the insulin resistance, a clinical condition that precedes for many years the appearance of T2D. In keeping with this, some studies have suggested that there may be an association between insulin sensitivity and arterial stiffness in individuals without diabetes [21], as well as in young healthy women [22], diabetic adults [23], and in nondiabetic hypertensive older adults [24]. This association may be explained by the fact that insulin resistance is associated with reduced nitric oxide bioavailability [25] that regulates local arterial distensibility; in addition, hyperinsulinemia may contribute to the proliferation of arterial smooth muscle [26]. These factors may lead very early to arterial stiffness before the development of impaired glucose tolerance or diabetes. In keeping with this, there are several mechanisms by which arterial stiffness increases in IGT or diabetic individuals. It’s well-known that chronic hyperglycemia induces non-enzymatic glycation of both circulating proteins and those present in the cellular membrane, leading to the formation of advanced glycation end products (AGEs). When AGEs accumulate in the arterial wall, they make irreversible and stable links with collagen polymers, leading to fibrosis development with increase of PWV and aortic hemodynamic parameters [27]. Diabetes also promotes increased lipid oxidation, vasoconstriction, tissue remodeling, mild vascular inflammation, and sympathetic nervous system activation, all factors associated with the initiation and progression of atherosclerosis [27], [28]. Finally, it is important to remark that some of the mechanisms by which arteries stiffen after exposure to elevations in plasma glucose occur rapidly, such as endothelial dysfunction that deteriorates during both postprandial hyperglycemia and hypertriglyceridemia [29], [30]. Thus, the most clinically relevant information, from this study, is that all these pathological modifications of vascular wall begin early, even in NGT state that could be considered a false condition at very low cardiovascular risk.

The prognostic importance of PWV has been attributed to adverse hemodynamic effects of aortic stiffening, characterized by an increase in both systolic BP and PP with increased systolic load and decreased myocardial perfusion pressure. Importantly, this study also demonstrates a strong relationship between plasma glucose concentration and arterial stiffening independently of other known risk cardiovascular factors. Thus, it appears clinically relevant that impaired glucose regulation, characterised in particular by post-challenge hyperglycaemia and insulin resistance, may be considered a readily treatable example of an alternative pathway promoting vascular stiffness. In keeping with this, present data have allowed to identify a new early predictor of organ damage and emphasize the importance to perform an OGTT in all subjects affected by essential hypertension, paying attention not only to 2- h but also to 1-h post-load plasma glucose value, which is more strongly associated with PWV and aortic hemodynamic parameters.
